# *The Organon* and Materia Medica Club of the Bay Cities of California

**Published:** 1897-08

**Authors:** W. E. Ledyard

**Affiliations:** B. A., M. B., M. R. C. S. Eng., Secretary


### *THE ORGANON* AND MATERIA MEDICA CLUB OF THE BAY CITIES OF CALIFORNIA.

The regular semi-monthly meeting was held at the office of Dr. George H. Martin, 606 Sutter Street, San Francisco, on Friday evening, February 19th, 1897.

Members present: Drs. J. M. Selfridge, George H. Martin, and W. E. Ledyard.

The meeting was called to order at 8 o'clock by the President, Dr. J. M. Selfridge.

The minutes of the two preceding meetings were read by the Secretary; corrected and approved.

The President appointed Dr. Ledyard to fill the vacancy left *pro tem.* in the Board of Censors by the absence of Dr. Wilson.

The Censors reported favorably on the name of Dr. Dyer.

The President then declared Dr. Dyer elected as a senior member of the club.

*The Organon* was then read and discussed from Section 284 to the end.

*Dr. J. M. Selfridge*, referring to note to Section 287, said: "If Hahnemann had understood the molecular theory of matter he would have known why potentizing medicines makes them more powerful curatively. The reason is that potentizing sets free the molecules."

*Dr. Martin* said: "In discussing this part of *The Organon*, as an illustration, I always think of the old practice of swallowing a bullet to move the bowels. The lead of the bullet acts simply by its mechanical effect, while if triturated it would kill."

*Dr. Ledyard*, to illustrate Section 288, which refers to the "rapid spread through the organism of the effect of medicines in liquid form," said: "Although we can conceive that a serpent poison can spread through the system with great rapidly, yet, judging from analogy, we should say that the effect of the same highly potentized poison will act with inconceivable rapidity on the same parts, and thus completely antidote or remove the effects of the former. We have observed this in a case of bee-sting, in which the 1,000th potency of Apis almost instantaneously completely removed the effects of the sting. Also a case of poisoning by strong ammonia, in which Swan's CM potency of Ammonia-carb. relieved in a few minutes.

*Dr. J. M. Selfridge*—"The antidotal use of the same medicine highly potentized seems very reasonable."

On motion it was decided that the Club (having finished the reading and discussion of Hahnemann's *Organon* for the second time), at the next meeting commence the reading and discussion of Hahnemann's *Chronic Diseases.*

The Club then adjourned to meet at the office of Dr. Selfridge, 1068 Broadway, cor. Twelfth Street, Oakland.

W. E. LEDYARD, B. A., M. B., M. R. C. S. Eng.,
*Secretary.*

---

